# Frizzled-7 promoter is highly active in tumors and promoter-driven Shiga-like toxin I inhibits hepatocellular carcinoma growth

**DOI:** 10.18632/oncotarget.5516

**Published:** 2015-10-16

**Authors:** Hongpan Xu, Lailing Gong, Yanyan Xia, Lili Qu, Qiwen Li, Lu Pang, Jin Si, Zhiyang Li

**Affiliations:** ^1^ Department of Laboratory Medicine, the Second Affiliated Hospital, Nanjing Medical University, Nanjing, Jiangsu 210011, China; ^2^ Department of Laboratory Medicine, Nanjing Drum Tower Hospital Clinical College of Nanjing Medical University, Nanjing, Jiangsu 210008, China

**Keywords:** Frizzled-7 promoter, pFZD7-GFP, Shiga-like toxin I, pFZD7-Stx1, gene therapy

## Abstract

Frizzled-7 protein plays a significant role in the formation of several malignant tumors. Up regulation of the Frizzled-7 in cancer cell lines is associated with nuclear accumulation of wild-type β-catenin from the Wnt/β-catenin pathway which is frequently activated in tumors. To analyze activity of the Frizzled-7 promoter in tumor cells, we constructed two recombinant plasmid vectors in which the Frizzled-7 promoter was used to drive the expression of green fluorescent protein (GFP) and Shiga-like toxin I (Stx1) (pFZD7-GFP/Stx1) genes. The Frizzled-7 protein was found to be expressed in the cancer cell lines but not in the normal cell lines. The GFP expression was restricted to the cancer cell lines and xenografts in the BALB/C mice but not to normal cell lines. Moreover, cell proliferation and tumor growth decreased significantly after transfection with the pFZD7-Stx1. Results from this study will help determine a highly effective strategy for gene therapy of tumors.

## INTRODUCTION

Cancer represents a leading health problem worldwide and surgery is not a treatment option in many cases, because of late diagnosis and poor therapeutic options available. Gene therapy represents an attractive approach for treatment of cancers and other chronic diseases. The optimal treatment for human cancer requires improvement of therapeutic ratio; specifically meaning that, treatment methods should be developed to maximize the cytotoxic efficacy of agents against tumor cells while minimizing their effect on normal cells [1, 2].

Members of the Frizzled family of seven-pass transmembrane proteins serve as receptors for the Wnt signaling glycoproteins. Activation of the Wnt/Frizzled signaling plays crucial roles during development of most neoplasms, such as hepatocellular carcinoma (HCC) and colon cancer [3]. The Wnts are a family of secreted glycoproteins that serve as extracellular signaling molecules involved in cell differentiation, migration, and proliferation during embryonic development. These proteins cause tumor formation when they are aberrantly activated [4–6]. Using reverse-transcriptase polymerase chain reaction (RT-PCR), researchers found that the Wnt mRNAs were over expressed in the HCC cells, where they detected the over expression of Wnt3, Wnt5A, Wnt6, and Wnt11 among the 19 studied human Wnt ligands [5].

Additional experiments revealed that the human Frizzled type 7 receptor (Frizzled-7) over expression stabilized the wild-type β-catenin and induced its translocation into the nucleus, the functional consequence of which was an increase in cell motility and migration. We therefore hypothesized in this study that the tumorigenesis of human cancer cell lines may be enhanced by the Wnt3 activity, which may lead to the aberrant activation of the Wnt/β-catenin signaling through the wild-type β-catenin protein [7–9]. Several successful studies have confirmed that the Frizzled-7 may be involved in enhancement of survival, invasion and metastatic capabilities of several types of human tumors [10–12] like colon, breast, pancreatic, lung and hepatocellular carcinoma. The enhancement has been confirmed to happen through the non-canonical Wnt and canonical signaling pathways.

Shiga-like toxin I (Stx1) is one of cytotoxic agents that can induce toxin-sensitive cells apoptosis through sequential activation of caspases, leading to chromatin condensation and DNA fragmentation [13]. It has been verified that the Stx1 is active against tumor cells in xenograft tumor models in mice [14, 15]. The Stx1 is produced by *Escherichia coli* pathogenic strains and consists of one A and five B subunits [16, 17]. Its nontoxic B-subunit binds to the cellular toxin receptor, glycosphingolipid globotriaosylceramide (Gb3), and is endocytosed [18, 19]. The Gb3 is highly expressed in human cancers, such as B-cell lymphomas [20], breast tumors [20], testicular seminomas [21] and colorectal carcinomas [22]. The A-subunit is translocated into the cytosol and inhibits protein biosynthesis by modifying rRNA [23]. The combination between the Frizzled-7 promoter and Stx1 may be used as a possible approach to augment the effect in a cancer gene therapy.

We in this study constructed two recombinant plasmid vectors in which the Frizzled-7 promoter was used to drive the expression of the green fluorescent protein (GFP) and Shiga-like toxin I (Stx1) genes (pFZD7-GFP/Stx1). We analyzed the Frizzled-7 protein and mRNA levels in various human cancer and normal cell lines and tissues using the Western blot, quantitative real-time PCR (qRT-PCR) and immunohistochemistry techniques. We transfected the normal liver and cancer cell lines with the pFZD7-GFP and pFZD7-Stx1 plasmids and conducted *in vitro* and *in vivo* experiments to analyze the influence of the Frizzled-7 promoter on cell cultures and living animals.

## RESULTS

### Construction of Frizzled-7 promoter and recombinant vectors

**Figure 1:**
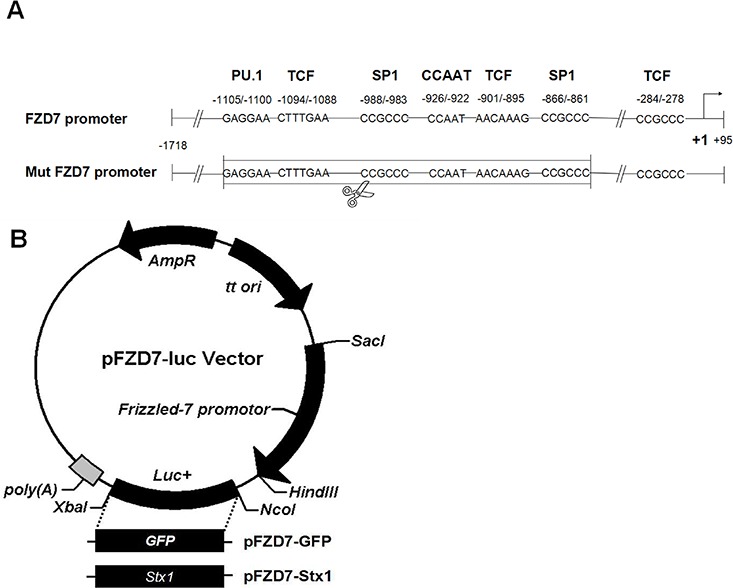
Construction of Frizzled-7 promoter and recombinant vectors **A.** The Frizzled-7 promoter comprised of nucleotides −1,718 to +95 (relative to Frizzled-7 transcription initiation site) was designed according to the DNA sequence of the human Frizzled-7 (accession number: AB017365). Several evolutionarily conserved transcription factor-binding sites, including PU.1-, SP1-, CCAAT box-, and TCF/LEF/SOX-, were all encompassed in the region of the Frizzled-7 promoter. The mutated Frizzled-7 promoter (Mut FZD7) was generated by deleting the predicted conserved region in the sequence. **B.** The pFZD7-GFP and pFZD7-Stx1 vectors were constructed by replacing the luciferase gene of pFZD7-Luc with the GFP or Stx1 genes using the *NcoI* and *XbaI* restriction endonucleases.

The Frizzled-7 promoter comprising of nucleotides −1,718 to +95 (relative to Frizzled-7 transcription initiation site) was designed according to the DNA sequence of the human Frizzled-7 (accession number: AB017365). Several evolutionarily conserved transcription factor-binding sites, including PU.1-, SP1-, CCAAT box-, and TCF/LEF/SOX-, were all encompassed in the Frizzled-7 promoter region [24, 25]. In addition, to further verify the Frizzled-7 promoter activity, we deleted the predicted conserved region to generate a mutated Frizzled-7 promoter (Mut FZD7) in the assay vector (Figure 1A). The Frizzled-7 promoter was cloned into the pGL3-basic vector using *SalI* and *HindIII* restriction endonucleases, generating a pFZD7-luc vector. The luciferase gene of pFZD7-Luc was replaced with the GFP (pFZD7-GFP) or Stx1 (pFZD7-Stx1) genes using the *NcoI* and *XbaI* restriction endonucleases (Figure 1B) and the fidelity of the plasmids was confirmed by sequencing analysis (data not shown). The control vector pFZD7 plasmid was constructed by deleting the pFZD7-Luc luciferase gene and the pGL3-GFP plasmid was generated by replacing the pGL3-basic luciferase gene with the GFP gene.

### RNA and protein levels of Frizzled-7 in human normal and cancer cell lines and rat tissues

**Figure 2:**
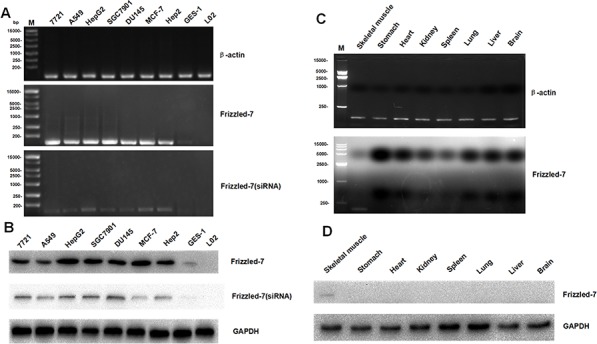
qRT-PCR and Western blot analyses of Frizzled-7 expression in cell lines and tissues from various species **A.** The expression levels of the Frizzled-7 RNA in human normal and cancer cell lines was shown by qRT-PCR (mean ± SD of three samples, relative to HepG2). The expression of Frizzled-7 RNA was significantly enriched in human cancer cells but not in L02 or GES-1 cells. The knockdown of the Frizzled-7 with siRNA led to a reduction in the expression of the Frizzled-7 RNA. **B.** The Frizzled-7 protein expression levels in human normal and cancer cell lines were shown by Western blot analysis. The protein were detected obviously in human cancer cells but not in L02 or GES-1 cells, and the siRNA reduced the expression of the Frizzled-7 protein expression. **C.** Frizzled-7 RNA expression in rat tissues was detected by qRT-PCR (mean ± SD of three samples, relative to Liver cancer tissues). The level of Frizzled-7 RNA was relatively low in rat normal tissues compared with rat liver cancer tissues. **D.** Frizzled-7 protein expression in rat tissues was detected by Western blot analysis. Weak Frizzled-7 protein expression was detected in the skeletal muscle of rat tissues, while the levels of Frizzled-7 expression were lower in other rat tissues. GAPDH expression was used as internal controls. All experiments were repeated more than three times.

RNA and protein extracts were prepared from the cells and tissues derived from different species and subjected to qRT-PCR and Western blot analyses. Figure 2A and Figure 2B showed that the human normal L02 (liver) and GES-1 (stomach) cell lines barely exhibited Frizzled-7 RNA and protein expression. The Frizzled-7 RNA and protein were over expressed in all the human cancer cell lines, including HepG2 (liver), 7721 (liver), A549 (lung), SGC7901 (stomach), MCF7 (breast), DU145 (prostate), and HEP2 (throat). To confirm the results from our study, a small interfering RNA (siRNA) was delivered to knock down the Frizzled-7 expression as a control. Consistent with our speculation, the knockdown of the Frizzled-7 with the siRNA led to a reduction in the expression of the Frizzled-7 (Figures 2A, and 2B). We also detected the Frizzled-7 expression in the rat tissues (Figure 2C, 2D). All of the normal rat tissues exhibited low expression levels of Frizzled-7 RNA expression (Figure 2C). In addition, a weak Frizzled-7 band was also detected in the rat tissues skeletal muscle while no band was detected in the other rat tissues as shown in Figure 2D.

### Immunohistochemistry analysis of *in vivo* Frizzled 7 protein expression

**Figure 3:**
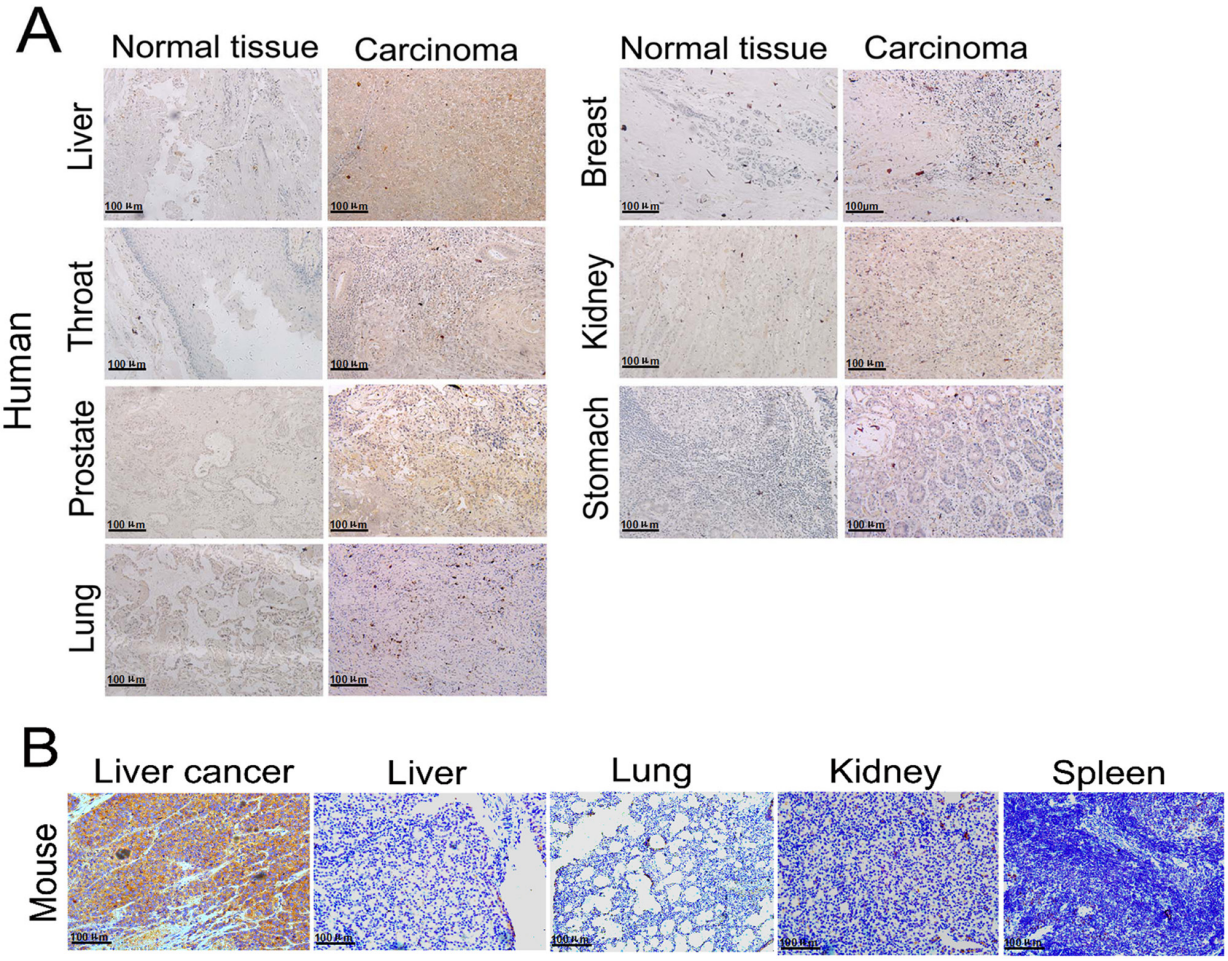
Immunohistochemical analysis of *in vivo* Frizzled-7 protein expression **A.** Immunohistochemical analysis showed over expression of Frizzled-7 protein in human tumor tissues, while there was no Frizzled-7 protein expression in the normal tissues (original magnification, 200 ×). **B.** Frizzled-7 protein was over expressed in liver tumor xenografts in nude mice (original magnification, 200 ×).

Human normal tissues (liver, throat, prostate, lung, breast, kidney and stomach) and corresponding carcinoma tissues were clinically obtained specimens from the archives of the Department of Pathology, the Second Affiliated Hospital of Nanjing Medical University. All the nude mice normal tissues and liver cancer tissues were taken from the experimental groups and the HCC xenografts were established by injection with 1 × 10^6^ HepG2 cells in serum-free media (100 μL) mixed with an equal volume of Matrigel in the right flank of the nude mice. All of the harvested tissues were fixed in 10% buffered formalin, embedded in paraffin, and sectioned with 6-μm thickness. The immunohistochemistry analysis was then used to detect the Frizzled-7 protein expression. We used a goat anti-rabbit Frizzled 7 polyclonal antibody and previously described indirect immunoperoxidase immunohistochemistry procedures to process and analyze the stained tissues. The immunohistochemistry analysis revealed that the Frizzled-7 protein expression was significantly reduced in the normal tissues derived from the human and nude mice while highly expressed in the tumors derived from the nude mice hepatocellular carcinoma cells and other human carcinoma tissues (Figure 3A and 3B).

### Frizzled-7 promoter's *in vitro* and *in vivo* activities

**Figure 4:**
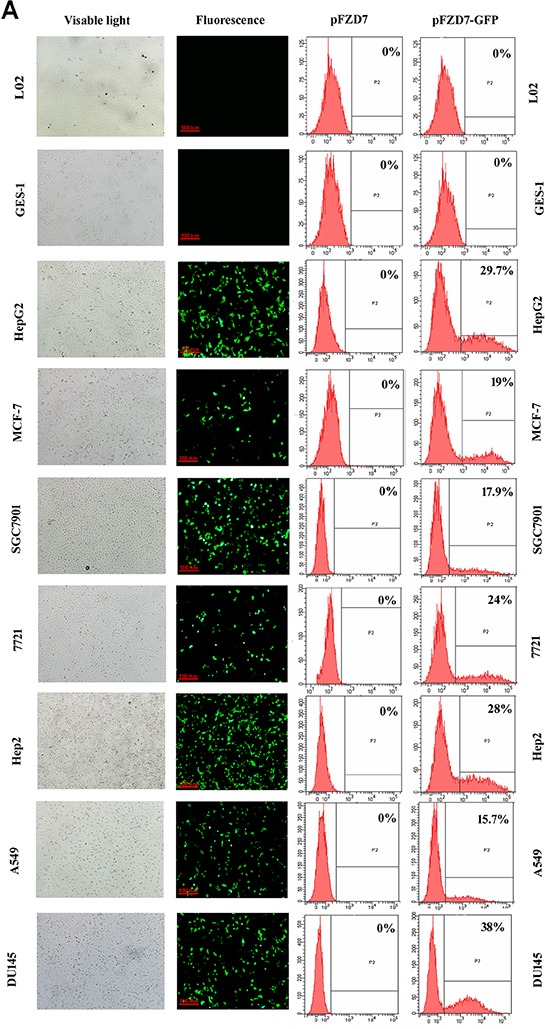

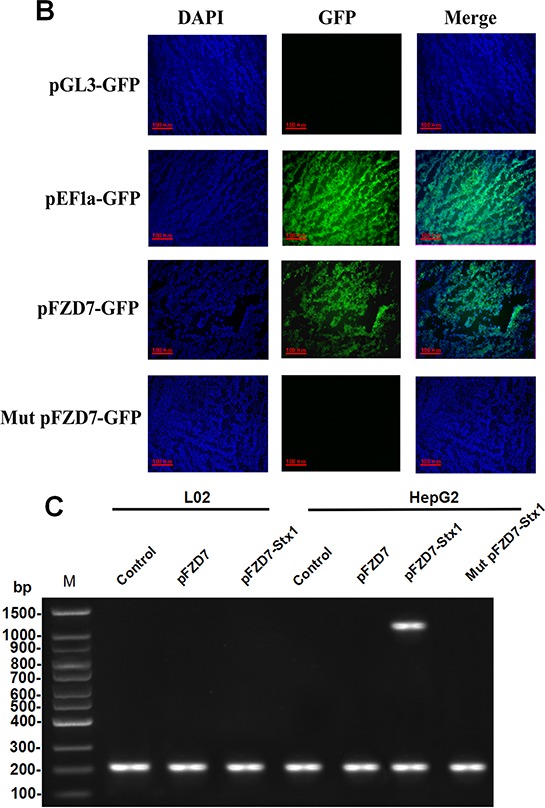
Activity of the Frizzled-7 promoter **A.** *In vitro* Frizzled-7 promoter activity in L02 and GES-1 cells and cancer cell lines after transfection with pFZD7-GFP, as observed by fluorescence microscopy (left) and detected by flow cytometry (right). The GFP gene was therefore over expressed in all the cancer cell lines in our study but not in the L02 and GES-1 cells. **B.** To determine the *in vivo* activity of the Frizzled-7 promoter, three control GFP plasmids, one with a mutated FZD7 promoter (Mut pFZD7-GFP), second with well-characterized EF1a promoter (pEF1a-GFP) and third without promoter (pGL3-GFP) respectively, were generated in the same way as pFZD7-GFP. Strong green fluorescence was detected in the frozen liver tumors tissue sections injected with the pFZD7-GFP and pEF1a-GFP (positive control). In contrast, the GFP activity was not detected in tissues injected with the Mut pFZD7-GFP and pGL3-GFP. The fluorescence from the liver cancer tissues was observed by fluorescence microscopy (original magnification 200 ×). **C.** RT-PCR analysis of *in vitro* Stx1 expression in HepG2 and L02 cells, as detected by agarose gel electrophoresis. The expression of Stx1 was only detected in HepG2 cells after transfection with pFZD7-Stx1, whereas the Stx1 gene expression was barely detected in the Mut pFZD7-Stx1 transfected HepG2 cells and pFZD7-Stx1 transfected L02 cells.

We employed a pFZD7-GFP reporter to determine the influence of the Frizzled-7 promoter on the human cancer cell lines HepG2 (liver), 7721 (liver), A549 (lung), SGC7901 (stomach), MCF7 (breast), DU145 (prostate), HEP2 (throat), and normal human cell line (L02, GES-1) after 48 h transfection. As shown in Figure 4A, the GFP was over expressed in the human hepatocellular carcinoma cell lines under control of the Frizzled-7 promoter, accounting for about 29.7% of all the cells by FCM analysis. Moreover, the GFP gene was over expressed in the 7721, SGC7901, A549, DU145, MCF-7, and Hep2 cells, where expression was noted in about 24%, 17.9%, 15.7%, 38%, 19%, and 28% of the listed cultured cells, respectively. In contrast, the GFP expression was not observed in the normal L02 (liver) and GES-1 (stomach) cells. The GFP gene was therefore over expressed (after induction by the Frizzled-7 promoter) in all the cancer cell lines in our study but not in the L02 and GES-1 cells (Figure 4A).

To determine the *in vivo* activity of the Frizzled-7 promoter, three control GFP plasmids, one with a mutated FZD7 promoter (Mut pFZD7-GFP), second with well-characterized EF1a promoter (pEF1a-GFP) and third without promoter (pGL3-GFP) respectively, were generated in the same way as the pFZD7-GFP. The pFZD7-GFP and three control plasmids were then intratumorally injected into the nude mice when their tumors reached a mean diameter of 5 mm. We detected strong green fluorescence in the frozen liver tumors tissue sections injected with the pFZD7-GFP and pEF1a-GFP as shown in Figure 4B, indicating that the Frizzled-7 promoter could drive the GFP expression *in vivo* as the EF1a promoter. In contrast, the GFP activity was not detected in tissues injected with the Mut pFZD7-GFP and pGL3-GFP. Also, similar results were found in the HepG2 cells transfected with the pFZD7-Stx1, whereas the Stx1 gene expression was also barely detected in the Mut pFZD7-Stx1 transfected HepG2 cells and pFZD7-Stx1 transfected L02 cells (Figure 4C).

### *In vitro* cytotoxicity and morphological changes induced by pFZD7-Stx1

**Figure 5:**
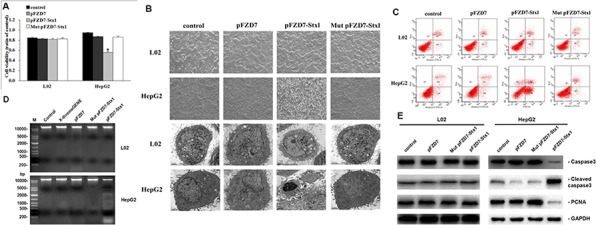
pFZD7-Stx1 transfection induced tumor cell apoptosis *in vitro* **A.** *In vitro* cytotoxicity from Stx1 on HepG2 and L02 cells, as detected by MTT assay. The relative cellular viability was measured by setting untreated cells as 100% cell viability. The HepG2 cells after pFZD7-Stx1-transfection showed significant decrease in cell viability when compared to the pFZD7-transfection (*p* < 0.01), Mut pFZD7-Stx1-transfection (*p* < 0.01) and untreated HepG2 cells (*p* < 0.01). There was no obvious cell viability decrease in the L02 cells after 48 h transfection with pFZD7-Stx1. Data are expressed as the mean ± SD of triplicate experiments. Error bars indicate SD. Where * indicates *p* value < 0.01. **B.** Morphological and ultrastructural changes of HepG2 and L02 cells after pFZD7-Stx1 transfection, as photographed under PCM at × 200 (upper panel) and TEM at × 5000 (lower panel). Obvious morphological changes and ultra structural apoptosis were observed through the light microscope and electron microscopy in the pFZD7-Stx1-transfected group but not in the other groups. **C.** Apoptosis in the HepG2 and L02 cells after pFZD7-Stx1 transfection, as detected by FCM. The HepG2 cells apoptotic necrosis rate, induced by the pFZD7-Stx1-transfecion, was significantly higher than that of the pFZD7-transfection, Mut pFZD7-Stx1-transfection and other control groups (*p* < 0.01). **D.** DNA fragmentation in the HepG2 and L02 cells after pFZD7-Stx1 transfection, as detected by agarose gel electrophoresis. The pFZD7-Stx1-transfecion could induce the DNA fragmentation in the HepG2 cells but not in the other groups from the HepG2 and L02 cells. **E.** Western-blot analysis of apoptosis and proliferation signaling in HepG2 and L02 cells after pFZD7-Stx1 transfection. The total caspase 3 and PCNA expression in the pFZD7-Stx1-transfected group was lower than that from the other group cells, whereas the cleaved caspase 3 level of the pFZD7-Stx1-transfected group was higher than that of other groups. GAPDH served as a loading control. Each experiment was done in triplicate.

To verify whether the Stx1 driven by Frizzled-7 promoter could inhibit the hepatocellular carcinoma growth, the influence of the pFZD7-Stx1 on the hepatocellular carcinoma cellular proliferation and biochemical toxicity were assessed by the MTT assays. The HepG2 cells showed obvious decrease in cell viability 48 h after pFZD7-Stx1-transfection when compared to the pFZD7-transfection (*p* < 0.01), Mut pFZD7-Stx1-transfection (*p* < 0.01) and untreated HepG2 cells (*p* < 0.01), as shown in the cellular growth inhibition rate curves (Figure 5A). There was no obvious decrease in cell viability in the L02 cells after 48 h transfection with the pFZD7-Stx1. Moreover, no cellular proliferation difference was observed in the untreated, Mut pFZD7-Stx1-transfected and pFZD7-transfected HepG2 cells. In summary, the Stx1 showed selective inhibitory and obvious cytotoxicity effects on the hepatocellular carcinoma cell proliferation when compared with the normal liver cells. A large number of HepG2 cells displayed more obvious morphological changes 48 h after transfection with the Stx1 under the light microscope (Figure 5B). Ultra structural apoptosis characteristics, including chromatin condensation and nuclear fragmentation, were also observed through the electron microscopy in the pFZD7-Stx1-transfected group as shown in Figure 5B. There were no obvious morphological and ultra structural changes in the HepG2 cells from the other three groups, and also in the L02 cells from all the other groups (Figure 5B). These findings demonstrated that the pFZD7-Stx1-transfection may have induced the apoptosis in the HepG2 tumor cells.

### *In vitro* cellular apoptosis induced by pFZD7-Stx1

The aim of this part of the study was to prove that the pFZD7-Stx1-transfection kills tumor cells or induce cellular apoptosis through some pathway. The FCM analysis was used to confirm this, as a useful method for testing the apoptosis. The FCM was applied to quantify the extent of apoptosis based on Annexin V-FITC and PI staining. The HepG2 cells apoptotic necrosis rate, as induced by the pFZD7-Stx1-transfecion, was significantly higher than that of the pFZD7-transfection, Mut pFZD7-Stx1-transfection or untreated HepG2 cells from the control group (*p* < 0.01), as demonstrated in Figure 5C. There were no significant differences between the apoptosis in the normal L02 cells transfected with the pFZD7-Stx1, pFZD7, Mut pFZD7-Stx1 or control group. The findings from the FCM analysis were confirmed by DNA fragmentation analysis. DNA fragmentation involves separation or breaking of the DNA strands into pieces and it is a natural fragmentation process that cells perform during apoptosis. As demonstrated in Figure 5D, the pFZD7-Stx1-transfecion could induce the DNA fragmentation in the HepG2 cells but not in the other groups of HepG2 and L02 cells. Caspase 3 and proliferating cell nuclear antigen (PCNA) are important downstream effecters of the cell apoptotic pathway. The total caspase 3, cleaved caspase 3 and PCNA protein levels were detected by Western-blot analysis, where the Glyceraldehyde-3-phosphate dehydrogenase (GAPDH) served as a loading control. As shown in Figure 5E, the total caspase 3 and PCNA expression in the pFZD7-Stx1-transfected group was lower than that from the other groups of cells, whereas the cleaved caspase 3 level of the pFZD7-Stx1-transfected group was higher than that of other groups. These findings strongly suggested that the Stx1 gene therapy system might have mechanistically repressed the caspase 3 and PCNA mediated cellular proliferation in the HepG2 (Figure 5E).

### pFZD7-Stx1 based gene therapy substantially reduced tumor burden and improved survival in a human HCC xenograft tumor from mouse model

**Figure 6:**
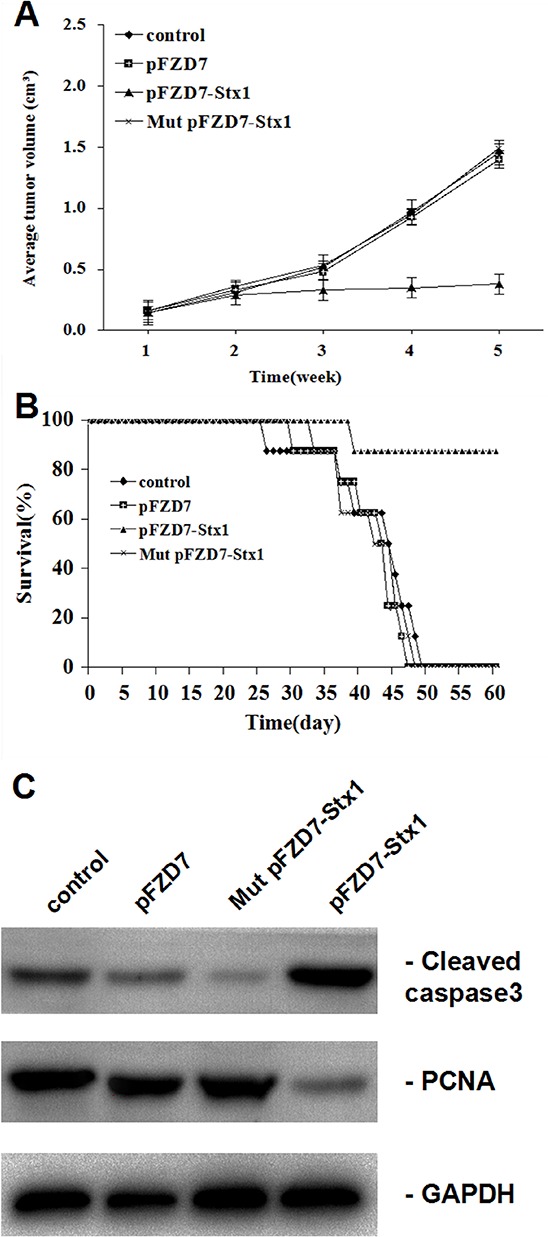
pFZD7-Stx1 reduced tumor burden and improved survival of nude mice with liver tumor xenografts *in vivo* **A.** The calculated average tumor volumes from the four groups at indicated time points. The average tumor volume after the pFZD7-Stx1 treatment was about 0.38 cm^3^ at 5 weeks compared with 1.46, 1.40 and 1.49 cm^3^ average tumor volumes after the PBS (*p* < 0.01), Mut pFZD7-Stx1 and pFZD7 treatments, (*p* < 0.01) respectively. Error bars indicate SD. **B.** The lifespan of xenografted mice after pFZD7-Stx1 treatment, as evaluated by the survival curves. 7 of the 8 mice in the pFZD7-Stx1-treated group were still alive 7 weeks post-injection of plasmids, which was in contrast to 1 of the 8 animals in the PBS, Mut pFZD7-Stx1 and pFZD7-treated groups. **C.** The levels of cell proliferation and apoptosis in liver tumor xenografts following different treatments. The level of cell PCNA in the pFZD7-Stx1-transfected group was lower than that of other group cells, whereas the cleaved caspase 3 expression in the pFZD7-Stx1-transfected group was higher than that in other groups.

The *in vivo* anti-tumor effect was evaluated in the liver tumor xenografts from a murine model. The tumor xenografts were established by subcutaneous injections with 1 × 10^6^ HepG2 cells in the serum free media (100 μL) in the right flank of the nude mice. These nude mice were used in the study about 10 d after injections. The pFZD7-Stx1 injected mice exhibited significant tumor growth suppression when compared with the PBS control and pFZD7-treated animals. The average tumor volume after the pFZD7-Stx1 treatment was about 0.38 cm^3^ at 5 weeks compared with 1.46, 1.40 and 1.49 cm^3^ average tumor volumes after the PBS, Mut pFZD7-Stx1 and pFZD7 treatments, (*p* < 0.01) respectively. These results were in agreement with results from the partial inhibition of the HepG2 human liver tumor growth (Figure 6A). The survival ratio was also significantly improved in the pFZD7-Stx1-treated mice as shown in Figure 6B. 7 of the 8 mice in the pFZD7-Stx1-treated group were still alive 7 weeks post-injection of plasmids, which was in contrast to 1 of the 8 animals in the PBS, Mut pFZD7-Stx1 and pFZD7-treated groups. The xenograft tumors were collected and measured levels of cell proliferation (PCNA) and apoptosis (active caspase3) following different treatments. As shown in Figure 6C, the level of the PCNA in the pFZD7-Stx1-transfected group was lower than that from the other groups of cells, whereas the cleaved caspase 3 expression in the pFZD7-Stx1-transfected group was higher than that in other groups. These results suggested that the pFZD7-Stx1 had a significant *in vivo* anti-tumor effect.

## DISCUSSION

A variety of promoters have currently been widely used in gene therapy for targeted cancer cells. However, they have some limitations for clinical use due to their low activity, poor specificity and potential toxicity to normal cells [26]. Previous studies have suggested that the Frizzled-7 promoter, whose expression is regulated at the transcriptional level [9], may control the transgene expression in cancer cells. The goal of this study was therefore to determine the relative activities/influences of the Frizzled-7 promoter in the lung, breast, stomach, prostate, throat and liver carcinomas, as well as L02 and GES-1 normal cells. Using the qRT-PCR and Western blot analyses, we provided direct evidence to confirm that the Frizzled-7 promoter is highly activated in tumor cells at the RNA and protein levels during the development of many cancers. On the other hand, we found out that the Frizzled-7 promoter was barely activated in the normal cell lines and most normal adult tissues. This expression pattern suggests a possible use of the Frizzled-7 promoter for gene therapy.

Previous studies have shown that the Frizzled-7 protein is universally expressed in the malignant tumors, such as colon cancer [10] and HCC [11]. The Frizzled-7 protein was detected as an intense band in the human cancer cell lines in our study, including the HepG2 (liver), 7721 (liver), A549 (lung), SGC7901 (stomach), MCF7 (breast), and DU145 (prostate) but not in the normal human cell lines and rat tissues. However, the findings from other studies also demonstrated that the over expression of the Frizzled-7 was observed not only in tumors but also in the matched surrounding dysplastic liver tissues [7, 9]. These observations may indicate that the over expression of the Frizzled-7 is an early event during the development of tumors [27, 28].

The GFP and Shiga-like toxin I were cloned into the PGL-3 basic non-viral vector pFZD7 in the current study and driven by the Frizzled-7 gene promoter. In contrast to the normal cell lines where the Frizzled-7 gene promoter is largely inactivated, the Frizzled-7 promoter in our study was ubiquitously activated in the cancer cell lines. The up regulation of the Frizzled-7, a Wnt3 receptor, is associated with activation of the Wnt/β-catenin cascade [7, 9]. The Wnt3 has been reported in the HepG2 (liver), A549 (lung), MKN45 (gastric), breast and rectal tumors [29], and it belongs to the Wnt1 class of ligands and canonical Wnt/β-catenin pathway. Previous studies found that the Wnt3 overexpression activates the Wnt/β-catenin pathway [5, 8, 30] and overexpression of the Frizzled-7 corresponds to the activation of the canonical Wnt pathway in the esophageal and gastric tumors [7, 8]. Aberrant accumulation of the β-catenin, which is the hallmark of the canonical Wnt signaling activation, is found in 30–70% of the HCCs and is not associated with mutations in the β-catenin, 5 Axin1, or adenomatous polyposis coli (APC) genes from the Wnt inducible signal transduction pathway [27, 31–33].

The β-catenin molecule is an important multifactorial protein which is involved in cell-cell adhesion by strengthening the linkage between cadherin and α-catenin in the actin cytoskeleton. Cytoplasmic and/or nuclear accumulation of the β-catenin is a frequent event in various human tumors, including colorectal, lung, breast, cervix, skin, and liver tumors [27, 28, 34–39]. Some studies have demonstrated that the aberrant activation of the Wnt/β-catenin signaling is associated with tumor development and progression [5, 40–43] and in agreement with this, we found in this study that the Frizzled-7 gene promoter could drive the GFP and Stx1 genes expression in the liver, lung, breast, gastric, throat, and prostate cancer cell lines. The Stx1 analysis showed that it could significantly decrease the HCC cell proliferation and suppress tumor growth after use of the pFZD7-Stx1. The apoptosis induced by the Stx1, a type-II ribosome-inactivating protein produced by the *Escherichia coli* pathogenic strains [44], was associated with enhanced expression of the pro-apoptotic protein Bax [45] and inhibited the expression of the anti-apoptotic Bcl-2 family member Mcl-1 [46]. When the Stx1 protein is endocytosed by the HCC cells, an enzymatically active part of the molecule subsequently enters the cytosol and efficiently inhibits protein synthesis, thereby killing the cells [47, 48].

Previous reports have also established that other promoters, such as the AFP, Rad51C, human telomerase reverse transcriptase (hTERT), and midkine promoters, are expressed in a majority of human cancers [49]. For example, the HCC and its promoter have been shown to drive the expression of exogenous therapeutic genes in tumors without influencing normal tissues but the percentage expression of the AFP, hTERT and midkine promoters in the HCCs was only 35%, 21.7% and 33% respectively, which was not high [50–53]. In contrast, previous studies have found that the Wnt3 and/or Frizzled-7 expression was up regulated in 60–90% of the human HCCs and 35–60% of the surrounding preneoplastic liver tissues, which also exhibited Frizzled-7 upregulation in comparison to normal liver tissue [5]. Consistent with these findings, our results demonstrated that the Stx1 driven Frizzled-7 promoter can inhibit the HCC growth without damaging the normal liver cells.

Although evidence has been building that Frizzled-7 is an important cell surface receptor governing Wnt signaling in cancer cells and accelerating cancer development and progression, Frizzled-7 is not exclusively expressed in cancer tissue but is also expressed and plays an important role in stem cells, such as embryonic stem cells and LGR5+ colonic crypt cells [54, 55]. Frizzled-7 can drive the symmetric expansion of satellite stem cells through the planar cell polarity pathway [56]. However, the level of Frizzled-7 expression is relatively low in most normal mature cells. To a certain extent, Frizzled-7 may be an excellent marker to distinguish cancer versus normal tissue in cancer therapy. Of course, novel methods should be developed to distinguish between cancer cells and stem cells while using Frizzled-7 promoter to drive anti-tumor genes. We are studying a tumor targeting nanocarrier which will be used to combine with Frizzled-7 promoter in our future research.

Results from our current study thus generate great interest on the Frizzled-7 promoter, because it can efficiently drive the transgene expression in cancer gene therapy. It can also regulate the transgenes’ expression to limit the toxicity of therapeutic genes. We therefore believe that this study will facilitate additional advances in the rapidly developing gene therapy field.

## MATERIALS AND METHODS

### Cell culture

The following human cancer cell lines (tissue of origin provided in parentheses) were used in this study: HepG2 (liver), 7721 (liver), A549 (lung), SGC7901 (stomach), MCF7 (breast), DU145 (prostate), and HEP2 (throat). The normal cell line used in this study was L02 (liver) and GES-1 (stomach). All the cells were provided by our laboratory and the tissues of origin were isolated from adult rats which were purchased from the Experimental Animal Center of Nanjing Medical University. The cells were cultured in Dulbecco's Modified Eagle's Medium (DMEM) plus 10% fetal bovine serum (FBS) (GIBCO-BRL), 100 U/mL penicillin, and 100 μg/ml streptomycin at 37°C in a humidified atmosphere containing 5% CO_2_.

### Plasmid cloning

Three different plasmids were generated in this study. The luciferase expression plasmid under the control of the Frizzled-7 promoter (FZD7-Luc) was cloned using a pair of primers: (5′-GAGCTCCAAATGGTTGCTTCTGC-3′ and 5′-TGCACTCGCGGCCGGCGAAGCTT-3′), designed according to the DNA sequence of the human Frizzled-7 (accession number: AB017365) promoter. The Frizzled-7 promoter was then amplified by polymerase chain reaction (PCR) which was performed using human bacterial artificial chromosome libraries (ResGen, Inc.) as a template. The 1813-bp segment of PCR products were digested by *ScaI* and *HindIII* and subcloned into the *ScaI*-*HindIII* site of the pGL-basic vector. To generate the pFZD7-GFP and pFZD7-Stx1 plasmids, the luciferase gene from the pFZD7-luc was replaced with GFP or mutated Stx1 genes. The control pFZD7 vector was constructed by deleting the luciferase pFZD7-luc gene. These plasmids were confirmed by restriction endonuclease digestion and sequencing analysis.

### RNA interference and transfection

The siRNA (Invitrogen, USA) targeting FZD7 was constructed according to human *Frizzled-7* sequence (accession number: AB017365). Transfections were performed with Lipofectamine RNAiMAX reagent (Invitrogen, USA) according to the manufacturer's protocol. Cells were seeded to be 60–80% confluent (0.25–1 × 10^6^) per well in a six-well dish and transfected with 10 μM siRNA for 48 hours with transfections. The stable clones were cultured in a medium for subsequent experiments.

### RNA isolation and quantitative real-time PCR

RNA was isolated from the cell lines or frozen tissue blocks using the RNA isolation reagent Trizol (Abcam) according to the manufacturer's instructions. The quality of the RNA samples was determined by electrophoresis through agarose gels and staining with GelRed (Biotium, USA). The RNA was treated with DNAse-free to eliminate any contaminating DNA. Total cDNA was synthesized from 1 μg total RNA in a 20 μL reaction volume with 1 μL of the oligo(dT)_18_ primer (0.5 μg/μL) and 1 μL of 200 U/μL RevertAid M-MuLV reverse transcriptase according to the manufacturer's instructions. One microliter of the cDNA sample was taken for the amplification of different transcripts using various primer sets. The amplification conditions were as follows: 95°C for 3 min; followed by 35 cycles of 94°C for 3 s, 55°C for 30 s, 72°C for 45 s; and a final extension of 72°C for 10 min. The PCR reactions were carried out in a 20 μL volume in the presence of 1 μL of each of the forward and the reverse primers using 0.05 U/μL of Taq polymerase according to the kit instructions (Thermo). The primer sequences used were as follows: Frizzled-7 gene: (sense) 5′-GTGCCAACGGCCTGATGTA-3′ and (antisense) 5′-AGGTGAGAACGGTAAAGAGCG-3′; β-actin: (sense) 5′-TGACGTGGACATCCGCAAAG-3′ and (antisense) 5′-CTGGAAGGTGGAC AGCGAGG-3′. The relative expression levels were measured by qRT-PCR. Ct values were obtained with Bio-Rad CFX Manager software and Delta Ct values were calculated, with a final analysis done in MS Excel (Microsoft). Each of the experiments was performed in triplicate.

### Western blot analysis

Cells were harvested with trypsin/EDTA and PBS-washed cell pellets stored at −70°C until use. Whole-cell protein was separated by sodium dodecyl sulfate-polyacrylamide gel electrophoresis. Protein was extracted from normal tissues (brain, heart, lung, liver, stomach, small intestine, bladder, kidney, ovary, oviduct, uterus, spleen, and skeletal muscle) of female SD rats. Tissues were homogenized in Mini bead beater and the TMER tissue protein extraction regent. 10 μg of total protein extract was resolved on a sodium dodecyl sulfate-12% polyacrylamide gel and transferred to nitrocellulose membranes for Frizzled-7 detection. The blots were hybridized with an anti-Frizzled-7 polyclonal antibody (diluted 1:1000; Abcam), followed by incubation with a peroxidase-conjugated goat anti-rabbit secondary antibody (diluted 1:1000; SigmA). Specificity of the anti-Frizzled-7 polyclonal antibody was confirmed by Western blot analysis with specific FZD7 peptide (Supplementary Figure S1). The peroxidase activity was detected by the ECL (GE Healthcare) method. This assay was performed in triple.

### Immunohistochemical analysis

Human normal tissues (liver, throat, prostate, lung, breast, kidney and stomach) and corresponding carcinoma tissues were clinically obtained from the archives of the Department of Pathology, the Second Affiliated Hospital of Nanjing Medical University. All the nude mice normal tissues and liver cancer tissues were taken from the experimental groups. The HCC xenografts were established by injection with 1 × 10^6^ HepG2 cells in serum-free media (100 μL) mixed with an equal volume of Matrigel in the right flank of the nude mice. All of the harvested tissues were fixed in 10% buffered formalin, embedded in paraffin, and sectioned at 6-μm thickness. The immunohistochemistry analysis was then used to detect the Frizzled 7 protein expression. We used the goat anti-rabbit Frizzled 7 polyclonal antibody (diluted 1:1000, Abcam) and previously described indirect immunoperoxidase immunohistochemistry procedures to process and analyze the stained tissue.

### *In vitro* Frizzled 7 promoter activity

The pFZD7-GFP plasmid was transiently transfected into the tumor cell lines using the X-tremeGENE HP DNA Transfection Reagent (Roche, USA). The HepG2 (liver), 7721 (liver), A549 (lung), SGC7901 (stomach), MCF7 (breast), DU145 (prostate), and HEP2 (throat) cells were then transfected with the pFZD7-GFP plasmid. Briefly, 3 × 10^5^ cells were placed into each well of a six-well plate in 2 mL of complete medium. 60–70% confluence was achieved after the cells were incubated overnight. A mixture of 2 μg of pFZD7-GFP plasmid DNA and control vector pFZD7 plasmid DNA was then obtained. 6 μL of the transfection reagent and 200 μL of serum-free medium were then added into each well. The plasmids transfection efficiency from all cells was detected by fluorescence microscopy and FCM forty-eight hours after transfection. Each assay was performed more than three times.

### *In vivo* Frizzled 7 promoter activity

Female BALB/c nude mice (age, 4–5 weeks) were purchased from the Experimental Animal Center of Nanjing Medical University. All rats were maintained under specific pathogen-free conditions at constant temperature (24°C ± 2°C) and humidity (50% ± 5%). They were then exposed to a 12-hour light/dark cycle with free access to commercial food and sterile water. Tumor xenografts were established by injection with 1 × 10^6^ HepG2 cells in serum-free media (100 μL) mixed with an equal volume of Matrigel in the right flank of the nude mice. The nude mice were utilized for further studies when the tumors reached a 5 mm mean diameter. This animal study was performed according to institutional guidelines conformed to the National Institutes of Health guidelines on the ethical use of animals.

To analyze the *in vivo* Frizzled-7 promoter activity, the nude mice were injected with 100 μL of the mixture containing 20 μL of the pFZD7-GFP and cationic lipids at three different points in the right flank every 4 days. The control mice were injected with normal saline and pEF1a-GFP, Mut pFZD7-GFP or pGL3-GFP plasmids with cationic lipids. The mice were sacrificed and tumors removed from them after two rounds of injection. Tissues dissected from the tumors were embedded with OCT embedding compound and 5-μm-thick sections were prepared. The tumor sections were then fixed with 4% paraformaldehyde and visualized by immunofluorescence.

### Stx1 expression analysis

The Stx1 expression was evaluated using the real-time RT-PCR using the total RNA isolated from the transfected HepG2 and L02 cells. Briefly, the HepG2 and L02 cells were plated in triplicate into the 6-well plates at 3 × 10^5^ cells/well density and cultured routinely for 24 h. The cells were then transfected with plasmids using Lipofectamine™ 2000. The cells were later harvested 48 h by trypsin digestion. The transfection of L02 cells was confirmed effective (Supplementary Figure S2). Total cellular RNA was extracted using Trizol reagent (Invitrogen). The primers used for Stx1 were; 5′-TAGGGATCCATGAAGATAATTATTTTTAGAG-3′ (sense) and 5′-ATCCTCGAGCGTCAACGAAAAAT AACTTCGCTGAATCCCCCTCCATTATG-3′ (antisense), and for β-actin; 5′-TGACGTGGACATCCGCAAAG-3′ (sense) and 5′-CTGGAAGGTGGACAGCGAGG-3′ (antisense), respectively. The Stx1 mRNA level was analyzed by one-step real-time RT-PCR using the RNA-direct™ SYBR Green Real time PCR Master Mix (Toyobo), according to the manufacturer's instructions. The cycling conditions were: 90°C for 30 s, 61°C for 20 min, 95°C for 60 s, then 40 cycles at 95°C for 15 s, 60°C for 1 min. The Stx1 mRNA level from each sample was normalized to that of the β-actin mRNA. The amplification was monitored on an ABI prism 7500 real-time PCR apparatus (Applied Biosystems). Non-reactivity of the primers and probe with contaminating genomic DNA was tested by the inclusion of controls that omitted the reverse transcriptase from the cDNA synthesis reaction (no RT controls). This experiment was done in triplicate.

### Cellular proliferation assay

Cellular proliferation was analyzed by 3-(4, 5-dimethylthiazol-2-yl)-2, 5-diphenyltetrazolium bromide (MTT, Sigma, USA). Briefly, 8000 cells/well from each group were plated into three 96-well microplates in 200 μL of medium. The cells from each microplates were next day transfected with different plasmids using *X-tremeGENE HP* DNA Transfection Reagent (Roche, USA), according to the instructions by the manufacturer. 50 μL of the MTT substrate was then added into each well after 24 h, 48 h and 72 h, respectively. The plates were then returned to standard tissue incubator conditions for additional 4 h. The medium was then removed and cells solubilized in 150 μL of dimethyl sulfoxide. The colorimetric analysis was performed at 550 nm wavelength and inhibition rate calculated as 1-(OD value of the transfection/OD value of untreated HepG2) × 100%. Each experiment was done in triplicate.

### Morphology and ultrastructural analysis

The HepG2 and L02 cells were plated into a 6-well plate at 3 × 10^5^ cells/well density. The cells were then transfected with pFZD7-Stx1 or pFZD7 less than 24 h later and the untreated cells were used as control. The transfection of L02 cells was confirmed effective (Supplementary Figure S3). The morphological and ultrastructural changes were observed by the phase contrast microscopy and transmission electron microscopy (TEM), respectively. The cells were examined under a DMIRB phase contrast microscopy (Leica) after incubation for 48 h in DMEM plus 10% FBS (GIBCO-BRL).

Cells were fixed with 25 g/L glutaraldehyde in 0.1 mol/L of sodium cacodylate buffer and osmicated with 10 g/L osmium tetroxide for TEM analysis. The cell block was then stained, dehydrated in graded ethanol, infiltrated with propylene oxide, and incubated in a 60°C oven for 48 h overnight. The silver sections were cut with an Ultracut E microtome and collected on a carbon-coated grid, followed by staining with uranylacetate and Reynold's lead citrate. The sections were then examined under a TEM-1200EX (JEOL, Japan).

### Apoptosis detection by FCM

The apoptotic cells were also evaluated using annexin-V-fluorescein isothiocyanate (FITC) and propidium iodide (PI) Apoptosis Detection Kit (BD, USA), to examine early and late apoptotic changes as previously established. The samples were washed twice and adjusted to a 5 × 10^5^ cells/mL concentration with phosphate buffer saline (PBS). 200 μL of suspension was added into each labeled tube. 5 μL of annexin V-FITC and 10 μL PI (20 μg/mL) were then added into the labeled tube and incubated for at least 15 min at room temperature in the dark. 200 μL of cold PBS binding buffer was then added into each tube without washing and analyzed using the FCM analysis (BD, USA), as soon as possible (within 30 min). The apoptotic cells were defined as the population that was PI negative (indicating an intact plasma membrane) and Annexin V-FITC positive. This FCM assay was also done in triplicate.

### DNA fragmentation analysis

The cells (5 × 10^4^ cells) were centrifuged, re-suspended in 200 μL PBS and incubated with 4 μL RNase A for 0.5 h at 37°C. This was followed by digestion with 20 μL proteinase K for 1 h at 37°C, followed by lysis in 200 μL of lysis buffer (100 mmol/L Tris-HCl, pH 8.0) containing 0.2% Triton-X 100 and 1 mmol/L ethylene diamine tetraacetic acid (EDTA). The lysed cells were held at 70°C for 10 min and the solution was then mixed with 5 mol/L NaCl (20 μL) and isopropanol (120 μL), followed by incubation at −20°C for 24 h and centrifugation at 15000 g for 20 min. The precipitated DNA was dissolved in 5 μL of Tris-EDTA (TE) buffer and subjected to electrophoresis using a 1% agarose gel and Tris-acetate-EDTA (TAE) buffer at 100 V. The separated DNA fragments (DNA ladders) were visualized using a UV transilluminator. This assay was performed more than three times.

### Detection of apoptosis and proliferation signaling by western blot analysis

Cells were plated into the 6-well plates at 3 × 10^5^ cells/well density and incubated overnight. The cells from each microplates were next day transfected with the pFZD7-Stx1 or other plasmids using the *X-tremeGENE HP* DNA Transfection Reagent (Roche, USA) according to the manufacturer's instructions. Equal protein amounts were then separated by sodium dodecyl sulfate-polyacrylamide gel electropheresis (SDS-PAGE), 12% reducing gelsand transferred to polyvinylidene difluoride membranes (PVDF) (Millipore, Bedford, MA), followed by blocking with 5% non-fat milk in 10 mM Tris, pH 7.5, 150 mM NaCl, and 0.05% Tween 20 buffer (TBST), for 1.5 h at room temperature. The membranes were then probed with 1:1000 dilution of caspase 3 (Cell signaling, USA), cleaved caspase3 (Cell signaling, USA), proliferating cell nuclear antigen (PCNA, Santa Cruz, USA) or anti-glyceraldehyde-3-phosphate dehydrogenase (anti-GAPDH, Sigma, USA) antibodies, at 4°C overnight, followed by incubation in a 1:1000 secondary antibodies dilution. The protein bands were then detected using the ECL prime Western blotting detection reagent (GE, USA). All of the Western blots were performed at least 3 times.

### Animal studies

The tumor xenografts were established as mentioned above. 32 nude mice bearing the tumor xenografts were randomly divided into four groups (8 animals per group) and used for determining the *in vivo* pFZD7-Stx1 anti-tumor influence. The mice in the pFZD7-Stx1, Mut pFZD7-Stx1 and pFZD7 groups were directly injected with 100 μL of the mixture containing 20 μg of each plasmid and cationic lipids. The injections were given into the growing tumors from 3 directions twice a week for 3 weeks. The tumor sizes were then measured every week using sliding calipers. The tumor volume was calculated using the following formula: V = 1/2 × length × width^2^. The levels of cell proliferation and apoptosis following different treatments were measured by Western blot using the PCNA and cleaved caspase3 isolated from the tissues dissected from the tumors injected with pFZD7-Stx1, Mut pFZD7-Stx1, pFZD7 or PBS, using the same antibodies and procedures as mentioned above. The mice in the control group were injected with PBS only and survival was monitored and plotted against time after all the plasmid injections. The Animal studies performed conformed to institutional guidelines and the National Institutes of Health guidelines on the ethical use of animals.

### Statistical analysis

SPSS19.0 software was used for statistical analysis and each assay was performed at least three times. The data were expressed as mean ± SD. Student's *t*-test and ANOVA were used to determine the significance of differences in multiple comparisons and *P* < 0.05 was considered to be statistically significant.

## SUPPLEMENTARY FIGURES


