# Supplementary material for: Frizzled-7 promoter is highly active in tumors and promoter-driven Shiga-like toxin I inhibits hepatocellular carcinoma growth
Source: Oncotarget. 2015 Oct 16;6(37):39908–23. doi: 10.18632/oncotarget.5516 (PMC4741869; doi:10.18632/oncotarget.5516)
Supplement: Supplementary file 1 [file oncotarget-06-39908-s001.pdf]

SUPPLEMENTARY FIGURES

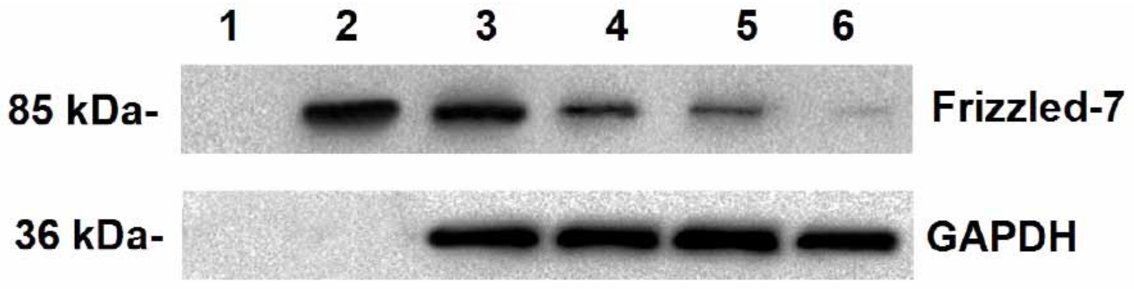

Supplementary Figure S1: Specificity of the anti-FZD7 polyclonal antibody was confirmed by Western blot analysis with specific FZD7 peptide. The blots of FZD7 peptide (Abcam, ab64635) and HepG2 sample incubated with the anti-FZD7 antibody as primary antibody exhibited an obvious 85 kDa bind. In contrast, when the anti-FZD7 antibody was incubated with the FZD7 peptide as a control antibody, the band was much weaker in the blot of HepG2 sample hybridized with the control antibody. In addition, the bind was not detected in the blot of L02 sample or the FZD7 peptide blot with application of secondary antibody alone. GAPDH served as a loading control. Each experiment was done in triplicate. (Lane 1: FZD7 peptide/secondary antibody alone; Lane 2: FZD7 peptide/ anti-FZD7 antibody; Lane 3: HepG2 sample/ anti-FZD7 antibody; Lane 4: HepG2 sample/ control antibody; Lane 5: L02 sample/ anti-FZD7 antibody; Lane 6: L02 sample/ control antibody).

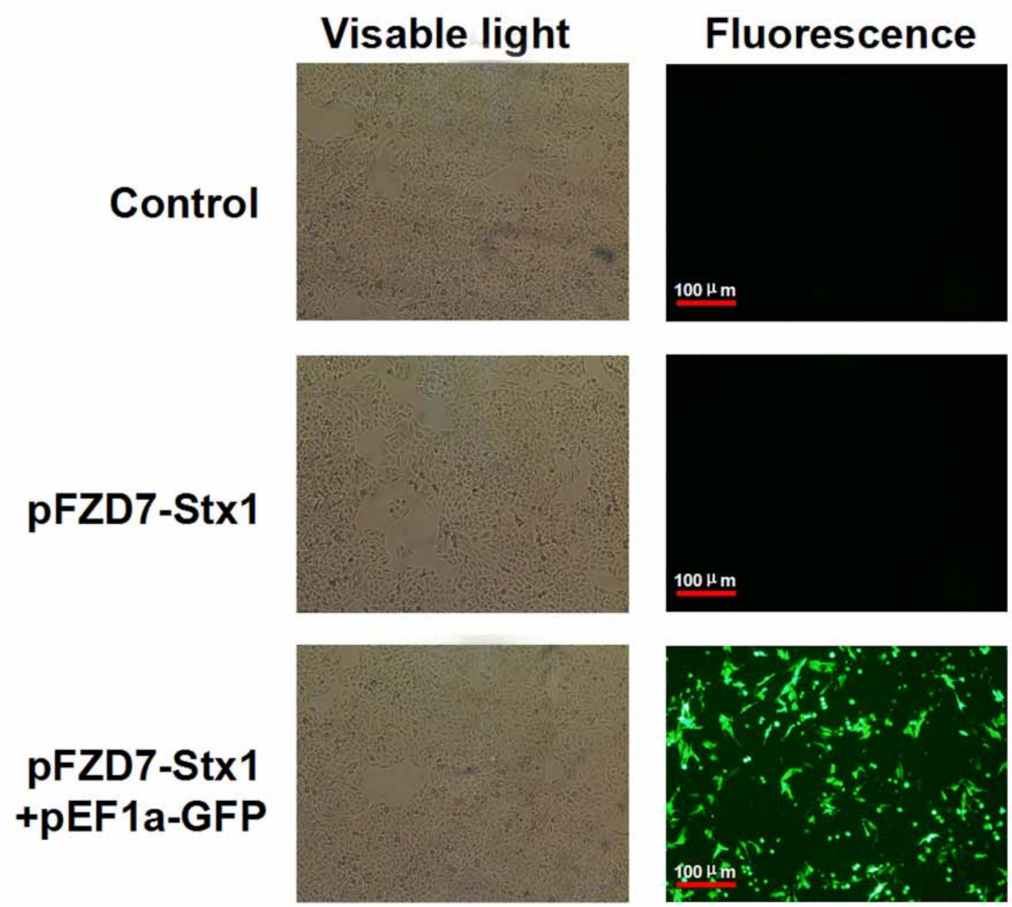

Supplementary Figure S2: The transfection of L02 cells was confirmed effective. We employed a pEF1a-GFP reporter to assess whether the transfection of L02 cells was effective. When L02 cells were transfected with pFZD7-Stx1 plasmid and pEF1a-GFP plasmid, we clearly detected the GFP activity through fluorescence microscopy (original magnification 200 ×). The GFP expression was not observed in the untransfected groups.

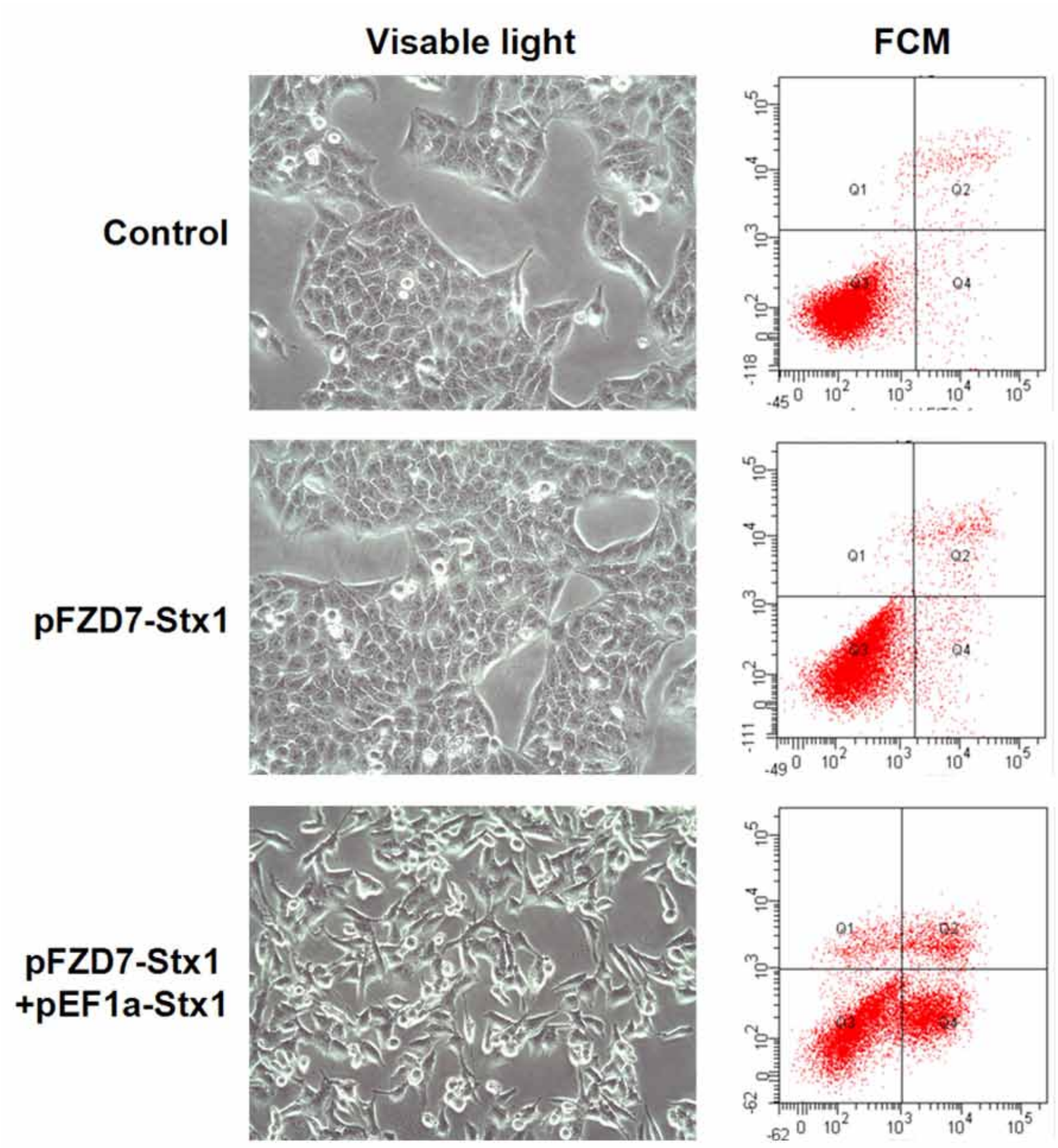

Supplementary Figure S3: L02 could be efficiently transduced with our plasmids and FZD7 promoter was not active in L02 cells. We constructed a pEF1a-Stx1 plasmid as pFZD7-Stx1. pEF1a-Stx1 plasmid was used to assess the transfection of L02 cells and compared with pFZD7-Stx1. The cytotoxicity effect was pretty clear when L02 cells were transfected with of pEF1a-Stx1 and pFZD7-Stx1, while there was not any obvious morphological changes and apoptosis in the L02 cells transfected with pFZD7-Stx1 alone as photographed (original magnification 400 ×) and FCM analysis.
